# Selectively Inhibiting the Median Preoptic Nucleus Attenuates Angiotensin II and Hyperosmotic-Induced Drinking Behavior and Vasopressin Release in Adult Male Rats

**DOI:** 10.1523/ENEURO.0473-18.2019

**Published:** 2019-03-26

**Authors:** Alexandria B. Marciante, Lei A. Wang, George E. Farmer, J. Thomas Cunningham

**Affiliations:** Department of Physiology and Anatomy, University of North Texas Health Science Center, Fort Worth, TX 76107

**Keywords:** ADH, Fos, osmoreceptor, thirst

## Abstract

The median preoptic nucleus (MnPO) is a putative integrative region that contributes to body fluid balance. Activation of the MnPO can influence thirst, but it is not clear how these responses are linked to body fluid homeostasis. We used designer receptors exclusively activated by designer drugs (DREADDs) to determine the role of the MnPO in drinking behavior and vasopressin release in response to peripheral angiotensin II (ANG II) or 3% hypertonic saline (3% HTN) in adult male Sprague Dawley rats (250–300 g). Rats were anesthetized with isoflurane and stereotaxically injected with an inhibitory DREADD (rAAV5-CaMKIIa-hM4D(G_i_)-mCherry) or control (rAAV5-CaMKIIa-mCherry) virus in the MnPO. After two weeks’ recovery, a subset of rats was used for extracellular recordings to verify functional effects of ANG II or hyperosmotic challenges in MnPO slice preparations. Remaining rats were used in drinking behavior studies. Each rat was administered either 10 mg/kg of exogenous clozapine-N-oxide (CNO) to inhibit DREADD-expressing cells or vehicle intraperitoneal followed by a test treatment with either 2-mg/kg ANG II or 3% HTN (1 ml/100-g bw, s.c.), twice per week for two separate treatment weeks. CNO-induced inhibition during either test treatment significantly attenuated drinking responses compared to vehicle treatments and controls. Brain tissue processed for cFos immunohistochemistry showed decreased expression with CNO-induced inhibition during either test treatment in the MnPO and downstream nuclei compared to controls. CNO-mediated inhibition significantly attenuated treatment-induced increases in plasma vasopressin compared to controls. The results indicate inhibition of CaMKIIa-expressing MnPO neurons significantly reduces drinking and vasopressin release in response to ANG II or hyperosmotic challenge.

## Visual Abstract

**Figure:**
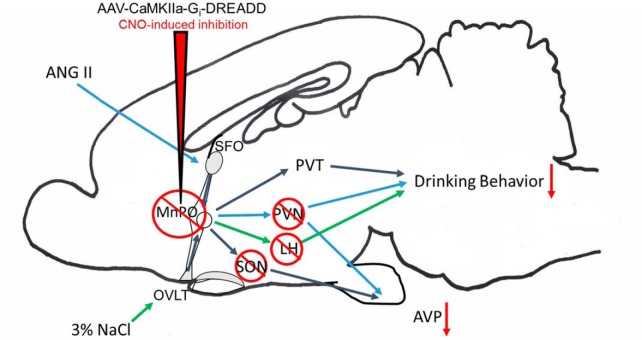

## Significance Statement

The median preoptic nucleus (MnPO) is an important regulatory center that influences thirst, cardiovascular and neuroendocrine function. Activation of different MnPO neuronal populations can inhibit or stimulate water intake. However, the role of the MnPO and its pathway-specific projections during angiotensin II (ANG II) and hyperosmotic challenges still have not yet been fully elucidated. These studies directly address this by using designer receptors exclusively activated by designer drugs (DREADDs) to acutely and selectively inhibit pathway-specific MnPO neurons, and uses techniques that measure changes at the protein, neuronal, and overall physiologic and behavioral level. More importantly, we have been able to demonstrate that physiologic challenges related to extracellular (ANG II) or cellular (hypertonic saline) dehydration activate MnPO neurons that may project to different parts of the hypothalamus.

## Introduction

The median preoptic nucleus (MnPO) is a midline nucleus that is part of the lamina terminalis, or anteroventral 3rd ventricle (AV3V) region. It plays an important role in receiving and integrating signals related to homeostasis and further propagating information to the hypothalamus (McKinley et al., 2015). Neurons in the MnPO contribute to the central regulation of sleep, core temperature, body fluid balance, hormone release, and autonomic function (McKinley et al., 2015). These studies focus on the role of the MnPO in regulating body fluid homeostasis through drinking behavior and vasopressin release.

The MnPO receives projections from two circumventricular organs (CVOs), the organum vasculosum of the lamina terminalis (OVLT) and the subfornical organ (SFO), that lie just ventral and dorsal to the MnPO along the anterior wall of the third ventricle, respectively. CVOs respond to fluctuations in plasma osmolality and other humoral factors, like angiotensin II (ANG II; Johnson et al., 1996). The OVLT and SFO project to several hypothalamic regions that contribute to homeostasis through behavioral, endocrine, and autonomic responses. These pathways include, but are not limited to, the supraoptic nucleus (SON), paraventricular nucleus of the hypothalamus (PVN), and the MnPO (Miselis, 1982; McKinley et al., 2004). The MnPO has reciprocal connections with the OVLT and SFO and also projects to the SON and PVN (McKinley et al., 2015). Studies have also shown that the MnPO has projections to the lateral hypothalamus (LH) and paraventricular thalamus (PVT) to regulate drinking behavior (Leib et al., 2017). The role, however, of the MnPO and its relationship to specific challenges regarding body fluid homeostasis are not completely understood.

Body fluid homeostasis involves orchestrated physiologic responses to cellular or extracellular dehydration (Fitzsimons, 1972; Adler and Verbalis, 2006). Cellular dehydration results from changes in body fluid solute content that is detected by osmoreceptors the in peripheral and central nervous system (Bourque, 2008). Extracellular dehydration is related to changes in body fluid volume that influence several hormonal and neural systems (Mecawi Ade et al., 2015; Dampney, 2016). Changes in the function of these systems contribute to several pathophysiological states (Adler and Verbalis, 2006). The contribution of the CNS to this physiology and pathophysiology has been the subject of previous investigations.

Lesions of the rat AV3V region produce a life-threatening adipsia (Johnson et al., 1996). If properly maintained, however, rats can recover some spontaneous water intake but do not respond to experimental challenges that mimic aspects of homeostatic thirst, such as ANG II or osmotic stimulation (Johnson et al., 1996).

Studies using electrolytic or chemical lesions of the MnPO have produced conflicting results. For example, electrolytic lesions of the MnPO are reported to increase baseline drinking and prevent vasopressin release (Gardiner and Stricker, 1985; Gardiner et al., 1985). Excitotoxin lesions of the MnPO inhibit experimentally-induced drinking behavior without affecting basal water intake (Cunningham et al., 1992).

A recent study by Allen et al. (2017) used a new technique involving the induction of Fos in neurons, a measurement for acute neuronal activation, co-transfected with TRAP creating an in-frame fusion to characterize MnPO neurons based on their activation by water deprivation or changes in body temperature. This study showed that putative thirst-related neurons in the MnPO are different from those that respond to body temperature. However, water deprivation is a complex, progressive physiologic challenge characterized by increased plasma osmolality, hypovolemia, and activation of the renin-angiotensin system. It is not clear whether MnPO neuronal activation associated with water deprivation is segregated by the physiologic stimulus or stimuli associated with water deprivation. The MnPO also contributes to other aspects of body fluid homeostasis in addition to thirst.

Recently developed chemogenetic and optogenetic techniques have been used to study neuronal circuitry responsible for mediating thirst in the lamina terminalis. A study by Augustine et al. (2018) recently used these approaches to characterize MnPO neurons that stimulate or inhibit thirst. Their results provide important information about the neurochemical phenotype of these two cell populations, however, the physiologic stimuli that regulate the activity of these cell types is not clear.

In the current study, AAV vectors with CaMKIIa promoters were used to express designer receptors exclusively activated by designer drugs (DREADDs) in the MnPO to inhibit CaMKIIa-expressing neurons with clozapine-N-oxide (CNO). Conscious rats were administered CNO during protocols that simulate extracellular (ANG II) or intracellular dehydration (hypertonic saline) to assess the role of the MnPO in behavioral and neuroendocrine responses to specific homeostatic challenges.

## Materials and Methods

### Animals

Adult male Sprague Dawley rats (250–300 g bw; Charles River Laboratories) were used for experiments. All rats were individually housed in a temperature-controlled (25°C) room on a 12/12 h light/dark cycle with light onset at 7 A.M. Food and water was available ad libitum except on the day of perfusions. Rats were weighed daily and their food and water intake monitored. Experiments were performed according to the National Institutes of Health guide for the care and use of laboratory animals and the Institutional Animal Care and Use Committee.

### Microinjection surgeries

Rats were anesthetized with 2% isoflurane and received stereotaxic microinjections of the inhibitory (AAV5-CaMKIIa-hM4D(G_i_)-mCherry) Cre-independent DREADD or control (AAV5-CaMKIIa-mCherry-Cre) virus (both from the UNC VectorCore) into the MnPO (microinjector angled at 8° from medial to lateral to avoid the septum, 0.0 mm anterior, 0.9 mm lateral, –6.7 mm ventral from bregma). A burr hole was then drilled at the measured site and a 30-gauge stainless steel injection needle was lowered to the MnPO, where 200–300 nl of AAV was delivered at a rate of 200 nl/min. The injector was connected to a Hamilton 5-µl syringe (#84851, Hamilton) by calibrated polyethylene tubing that was used to determine the injection volume. The injector remained in place for 5 min to allow for absorption and then slowly withdrawn. Gel foam was packed in to the drilled hole in the cranium. Absorbable antibiotic suture was used to close the incision site and minimize post-surgical infection. Each rat was given carprofen (Rimadyl, Bio-Serv, 1 mg) orally to minimize pain following surgery. Rats were allowed time for recovery and viral transfection for two weeks.

### *In situ* hybridization

*In situ* hybridization experiments were performed to characterize the neuronal phenotype of CaMKIIa-positive MnPO neurons transfected by the control virus. After the two-week recovery period, rats were anesthetized using 100-mg/kg inactin (Sigma-Aldrich) intraperitoneally and transcardially flushed first with RNase-free PBS. Rats were then perfused using 4% paraformaldehyde (PFA). Brains were dehydrated in RNase-free 30% sucrose. Twenty µm coronal sections of each brain were cut using a cryostat (Leica). Six sets of serial MnPO sections were collected in RNase-free PBS, mounted on to glass microscope slides, and left at room temperature overnight to dehydrate. Slides were then stored at –80°C until used for *in situ* hybridization experiments. *In situ* hybridization was performed for vesicular glutamate transporter 2, or vGLUT2 (RNAScope, Advanced Cell Diagnostics Inc.) using a previously established protocol (Smith et al., 2014).

### Electrophysiology

#### Slice preparation

Hypothalamic slices containing the MnPO were prepared as previously described (Farmer et al., 2018). Experimental animals were anesthetized with isoflurane and decapitated. Coronal slices (300 µm) containing the MnPO were cut using a Microslicer DTK Zero 1 (Ted Pella, Inc.) in ice cold (0–1°C), oxygenated (95% O_2_, 5% CO_2_) cutting solution consisting of 3.0 mM KCl, 1.0 mM MgCl_2_-6H_2_O, 2.0 mM CaCl_2_, 2.0 mM MgSO_4_, 1.25 mM NaH_2_PO_4_, 26 mM NaHCO_3_, 10 mM D-glucose, and 206 mM sucrose (300 mOsm, pH 7.4). Slices were incubated at room temperature in oxygenated (95% O_2_, 5% CO_2_) artificial CSF (aCSF) containing 126 mM NaCl, 3.0 mM KCl, 2.0 mM CaCl_2_, 2.0 mM MgSO_4_, 1.25 mM NaH_2_PO_4_, 26 mM NaHCO_3_, and 10 mM D-glucose (300 mOsm, pH 7.4) for a minimum of 1 h before recording.

#### Electrophysiology protocols

Slices containing the MnPO were transferred to a submersion recording chamber and superfused with aCSF (31 ± 1°C). Slices were visualized using an upright epifluorescent microscope (BX50WI, Olympus) with differential interference contrast optics.

Whole-cell (intracellular) recordings were performed in current clamp mode and conducted to measure whether CNO and DREADD-induced inhibition caused off-target effects that would influence the local circuitry. Recordings were obtained using borosilicate glass micropipettes (3–8 MΩ). The internal filling solution consisted of 145 mM K-gluconate, 10 mM HEPES, 1.0 mM EGTA, 2.0 mM Na_2_ATP, and 0.4 mM NaGTP (300 mOsm, pH 7.2). A tight gigaohm seal on MnPO neurons were made and had an access resistance of <25 MΩ. Neurons were slightly depolarized with current injection (2–5 pA) to generate a regular spiking activity (range, –50 to –40 mV), as previously described (Grob et al., 2004). Loose patch voltage clamp (extracellular) recordings were obtained using borosilicate glass micropipettes (1–3 MΩ) containing aCSF as the internal solution. Voltage was clamped at 0 mV to measure changes in current.

Electrophysiological signals (voltage and current) were amplified and digitized using Multiclamp 200B and Digidata 1440A, respectively (Molecular Devices). Signals were filtered at 2 kHz and digitized at 10 kHz. Recordings from MnPO neurons were made by targeting both mCherry-expressing and non mCherry-expressing neurons in slices prepared from rats injected with the AAV. Electrophysiological signals were analyzed using 10-s bins.

In the first set of experiments, intracellular or extracellular recordings were performed on MnPO neurons. Baseline membrane potential (for intracellular recordings) or action potential firing (for extracellular recordings) was recorded for 5 min. Then, CNO (10 µM) was focally applied for 10 s using a Pico spritzer (8 psi) with a patch pipette containing the drug placed 150–200 µm upstream of the recording electrode followed by an additional 10 min of recording. CNO (Tocris) was dissolved in DMSO and diluted in aCSF to final concentration of 10 µM (<0.01% DMSO).

In the ANG II experiments, baseline action potential firing was recorded for 5 min in either an aCSF bath solution containing CNO (500 nM) or aCSF alone (for G_i_ DREADD-labeled neuronal controls). Then, ANG II (10 µM) was focally applied for 10 s using a Pico spritzer (8 psi), as in the first set of electrophysiology experiments, followed by 10 min of recording. ANG II (A9525, Sigma-Aldrich) was dissolved and diluted in aCSF to a final concentration of 10 µM.

In the hyperosmotic challenge experiments, baseline action potential firing was recorded for 5 min in an aCSF bath solution containing CNO (500 nM). Then for 2 min, the bath solution was switched to CNO (500 nM) in hypertonic aCSF (HTN-aCSF; 330 mOsm) followed by 2 min of bath application of HTN-aCSF (330 mOsm). For the remaining 5 min of recording, aCSF was bath applied. Bath application of hyperosmotic solution was used instead of focal application because it allowed for a more exact control of the extracellular NaCl concentration that would not be possible with focal application. To control for G_i_ DREADD-labeled neuronal activity, experiments were performed following the same time course, however the protocol was 5 min in aCSF bath, 4 min in HTN-aCSF (330 mOsm), then 5-min aCSF bath. Additional NaCl was dissolved in aCSF to raise osmolality to 330 mOsm.

### Drinking study, ANG II

Before microinjections, rats were pretested with subcutaneous injection of ANG II twice, separated by 48–72 h, to determine individual drinking response to peripherally administered ANG II (consume ≥ 5 ml of water over the course of 3 h). Rats that responded were then microinjected with either the G_i_ DREADD or control virus and allowed recovery for two weeks. For each test, rats were intraperitoneally injected with 10-mg/kg CNO (dissolved in DMSO and diluted to the working concentration with 0.9% saline) or vehicle for CNO, VEH (DMSO and 0.9% saline in 1:4 ratio). Thirty minutes later, rats were administered ANG II (2 mg/kg, s.c., diluted to the working concentration in 0.9% saline) or the same volume of 0.9% saline. Water consumption was measured over the course of 3 h from the time ANG II was administered to measure drinking response and duration of CNO-induced inhibition. The substances injected for each test were administered in a randomized counter balanced order. The tests were separated by 48–72 h and were repeated two and three weeks after microinjection surgeries.

### Drinking study, 3% hypertonic saline (3% HTN)

A separate group of rats was used for drinking studies with 3% HTN. Rats were pretested consistent with those in the ANG II drinking study to determine individual drinking response to peripherally administered 3% HTN (consume ≥ 5 ml of water over the course of 3 h). Rats that responded were microinjected with the G_i_ DREADD or control virus and allowed recovery for two weeks. After the recovery period, animals were injected intraperitoneally with CNO or VEH and 3% HTN (1 ml/100-g bw) or the same volume of 0.9% saline. Study design and duration was consistent with the ANG II drinking study.

### Perfusions, tissue and body fluid collection

After the final treatment week for each drinking study, rats were administered intraperitoneally CNO and subcutaneously ANG II or 3% HTN, and denied food and water access the following 90 min. Animals were then anesthetized using 100-mg/kg inactin (Sigma-Aldrich) intraperitoneally. Blood was collected by cardiac puncture (3 ml) and transferred to an EDTA vacutainer containing 100 µl of aprotinin (catalog #RK-APRO, Phoenix Pharmaceuticals, Inc.) per milliliter of blood (300 µl total) immediately preceding the perfusion to measure plasma AVP and osmolality. Rats were transcardially flushed first with PBS and then perfused using 4% PFA. Brain tissue was fixed overnight in 4% PFA before being dehydrated in 30% sucrose.

### Arginine vasopressin

Plasma AVP concentrations were measured by specific EIA (Phoenix Pharmaceuticals, Inc.) following peptide extraction, as recommended by the manufacturer (catalog #EK-065-07, Phoenix Pharmaceuticals, Inc.). The volume of plasma used was 500 µl per sample for peptide extraction. A total of 100 µl from each sample was recovered from the extraction and assayed in duplicate (50 µl assayed). The total peptide concentration of each sample was calculated according to the directions provided by the manufacturer of the extraction kit. The intraassay and interassay coefficients of variation averaged <10% and <15%, respectively, as provided by the manufacturer (Phoenix Pharmaceuticals, Inc.).

### Immunohistochemistry

A total of 40-µm coronal sections of each previously perfused brain were cut using a cryostat. Three sets of serial sections were separately collected in cryoprotectant (Hoffman et al., 1992) and stored at –20°C until they were processed for immunohistochemistry. Separate sets of serial sections from brains injected with DREADD or control virus were stained for cFos (sc-253-G, goat polyclonal anti-Fos antibody, Santa Cruz Biotechnology, 1:1000). After 48 h, sections were washed using PBS and transferred to a secondary antibody (BA-9500, biotinylated anti-goat, Vector Laboratories) for DAB reaction and labeling. After the DAB reaction, the sections were washed and placed in the primary antibody (ab167453, rabbit polyclonal anti-mCherry, Abcam 1:500) and incubated for an additional 48 h followed by incubation with a Cy3-conjugated anti-rabbit antibody (711-165-152, Jackson ImmunoResearch) for 4–5 h. The sections were then mounted on gelatin-coated slides, dried, and coverslipped with Permount for imaging. All antibodies were diluted to the final concentration with PBS diluent (0.25% Triton X-100, 3% horse serum, and 96.75% PBS).

Sections were examined using light microscopy to identify Fos-positive cells. Excitation wavelengths of 550–570 nm were used for emission of mCherry immunofluorescence. Images were captured using an epifluorescent microscope (Olympus BX41, Olympus) equipped with a digital camera (Olympus DP70) to image sections. Care was taken to ensure that sections included in this study were sampled from the same plane in each brain section. Co-localization was determined by quenching produced in cells with nuclear fos staining and cytosolic mCherry staining, as previously described (Grindstaff et al., 2000). Brightfield and fluorescent images were merged for analysis of the MnPO using ImageJ (NIH). Fos-positive neurons and their co-localization in the MnPO was determined blind to experimental conditions of the subjects.

### Statistics

Electrophysiology data were tested for differences in baseline activity, changes in peak firing rate (defined as the 10-s bin with the lowest firing rate), and percentage change baseline firing rate using one-way ANOVA or two-way repeated measures ANOVA. Two-way repeated measures ANOVA was used to compare changes in water consumption between the G_i_ DREADD-injected group and control group and treatment (CNO vs VEH), followed by Tukey’s *post hoc* test. Analyses of plasma vasopressin concentrations, plasma osmolality, and cell counts for neuronal phenotyping were figured using one-way ANOVA followed by Tukey’s *post hoc* test. Tukey’s *post hoc* analysis was used when performing multiple comparisons. Holm–Sidak *post hoc* test was used to perform comparisons to a determined control. Statistical significance is defined at an α level of 0.05 and exact *p* values are reported. Values are reported as mean ± SEM. All statistics were performed in SigmaPlot.v.12.0 (Systat Software).

## Results

### CaMKIIa neurons in the MnPO signal through excitatory pathways

**Figure 1.**
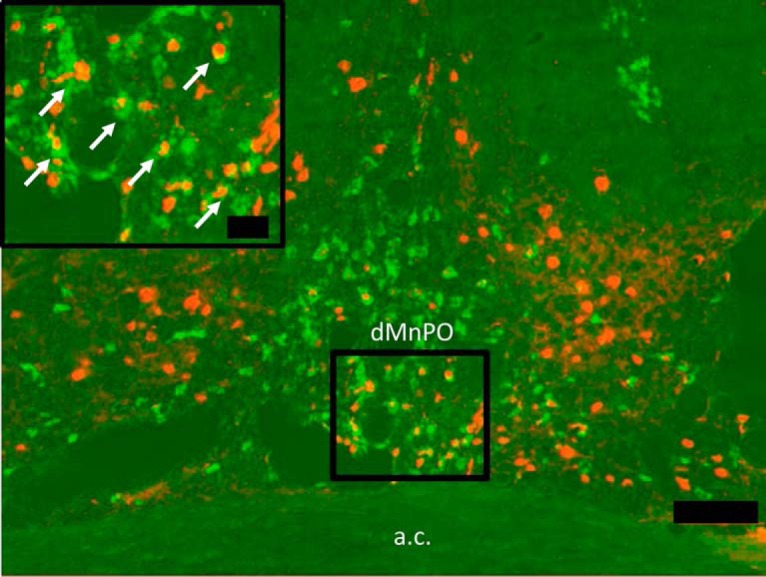
CaMKIIa-positive MnPO neurons are glutamatergic. Representative image of the dorsal MnPO (dMnPO) with CaMKIIa-positive MnPO neurons (red) and colocalization with vGLUT2 (green), as indicated in the inset by white arrows. Scale bar for inset, 25 µm. Main figure scale bar, 100 µm. Anterior commissure, a.c.

Experiments were conducted to determine the neurochemical phenotype of MnPO neurons that were targeted by the viral vectors with CaMKIIa promoters used in this study. *In situ* hybridization for vGLUT2 was used to identify putative excitatory MnPO neurons (*n* = 5 rats, two to three sections per rat). An example of vGLUT2 *in situ* hybridization and CaMKIIa immunohistochemistry in the dorsal MnPO is shown in Figure 1. The results show that 89.17 ± 1.32% of CaMKIIa neurons that express the viral construct co-localize with vGLUT2 message, indicating that the CaMKIIa-expressing neurons in the MnPO are primarily glutamatergic and would likely be involved in excitatory signaling.

### Electrophysiology results

#### CNO significantly attenuates basal firing rate in G_i_ DREADD-expressing neurons

##### Intracellular recordings

To verify that CNO was not having off-target effects, whole cell current clamp experiments (intracellular recordings) were conducted for more direct measurements on G_i_ DREADD (*n* = 5, three rats, two slices per rat) and G_i_ DREADDx (*n* = 7, four rats, two slices per rat) neurons within the same section. As previously described (Grob et al., 2004), neurons were slightly depolarized with current injection to generate a regular spiking activity (range, –50 to –40 mV) and allowed to stabilize in aCSF for 5 min before CNO exposure. There was a significant effect of treatment on neuron type (*F*_(3,20)_ = 21.175, *p* < 0.001, one-way ANOVA; data not shown). CNO exposure caused a significant membrane hyperpolarization in G_i_ DREADD neurons between aCSF baseline and peak response (–6.1 ± 0.9 mV; *p* = 0.001) but no significant change in membrane potential of the G_i_ DREADDx neurons in the presence of CNO compared to aCSF baseline (2.1 ± 0.9 mV; *p* = 0.979). These results indicate that CNO and the DREADD-induced inhibition of the G_i_ DREADD neurons did not have an effect on local circuitry.

##### Extracellular recordings

**Figure 2.**
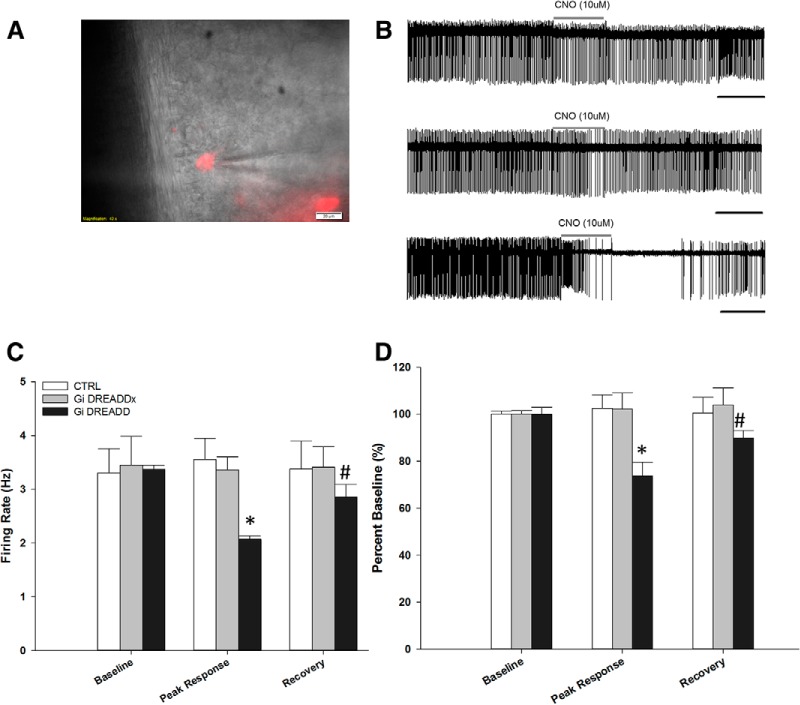
CNO-mediated inhibition significantly attenuates basal firing rate in G_i_ DREADD-labeled neurons. ***A***, Representative image showing loose-cell patch recording of a G_i_ DREADD-labeled (G_i_ DREADD) neuron (red). Scale bar, 20 µm. ***B***, Representative raw trace recordings of a control (CTRL) neuron (top, *n* = 13 neurons, six rats), G_i_ DREADD-unlabeled (G_i_ DREADDx) neuron (middle, *n* = 19 neurons, six rats), and G_i_ DREADD neuron (bottom, *n* = 17 neurons, six rats). Scale bar, 10 s. ***C***, CNO-mediated inhibition significantly attenuated basal firing rate of G_i_ DREADD neurons (peak response) compared to baseline and recovery, and compared to CTRL and G_i_ DREADDx. ***D***, CNO-mediated inhibition significantly attenuated basal firing rate of G_i_ DREADD neurons (peak response), represented as a percentage baseline, compared to G_i_ DREADDx and CTRL neurons; *p < 0.050, compared to peak response between groups; #*p* < 0.050, compared to baseline within group. Data are presented as mean and SEM.

Loose cell recordings were made from brain slices containing the MnPO two weeks after the rats were injected with AAVs containing either G_i_ DREADD or the control (CTRL) construct. Cells expressing the constructs were easily identifiable by expression of the mCherry reported (Fig. 2*A*). There were no differences in the rates of spontaneous activity of MnPO neurons transfected with either virus (G_i_ DREADD, 3.37 ± 0.1 Hz; *n* = 17, six rats, two slices per rat; CTRL, 3.30 ± 0.5 Hz; *n* = 13, six rats, two slices per rat) or unlabeled cells in slices from rats injected in the MnPO with the G_i_ DREADD (G_i_ DREADDx, 3.45 ± 0.5 Hz; *n* = 19, six rats, two slices per rat; *F*_(2,46)_ = 0.0306, *p* = 0.970, one-way ANOVA).

Focal CNO application (10 µM) significantly decreased the spontaneous activity of the G_i_ DREADD MnPO neurons (*F*_(1,32)_ = 16.22, *p* < 0.001, one-way ANOVA; Fig. 2*B*). When calculated as a percentage change from baseline, only G_i_ DREADD neurons showed a significant decrease in activity from baseline, or peak response (peak: defined as the 10-s bin with the lowest firing rate), that recovered after 172.4 ± 37.8 s (*F*_(2,46)_ = 10.050, *p* = 0.001, one-way ANOVA; Tukey’s *post hoc*, baseline vs peak *p* = 0.004 < 0.001 and baseline vs recovery, *p* = 0.024; Fig. 2*D*). There was also a significant difference in peak response to CNO in the G_i_ DREADD neuron firing rate (*F*_(2,46)_ = 10.832, *p* < 0.005, one-way ANOVA) compared to CTRL (*p* < 0.001) and G_i_ DREADDx neurons (*p* < 0.001) using Holm–Sidak *post hoc* test.

Analysis of the firing rates (Hz) of neurons from each group produced similar results (Fig. 2*C*). Focal CNO application did not significantly influence the spontaneous activity of the G_i_ DREADDx (*F*_(2,36)_ = 0.0808, *p* = 0.923, one-way ANOVA) or CTRL neurons (*F*_(2,54)_ = 0.0119, *p* = 0.988, one-way ANOVA; Fig. 2*D*). Additional experiments were therefore conducted to determine whether CNO could block changes in activity in CaMKIIa MnPO neurons produced by either ANG II or HTN.

### CNO blocks ANG II-induced excitation in G_i_ DREADD-expressing neurons

**Figure 3.**
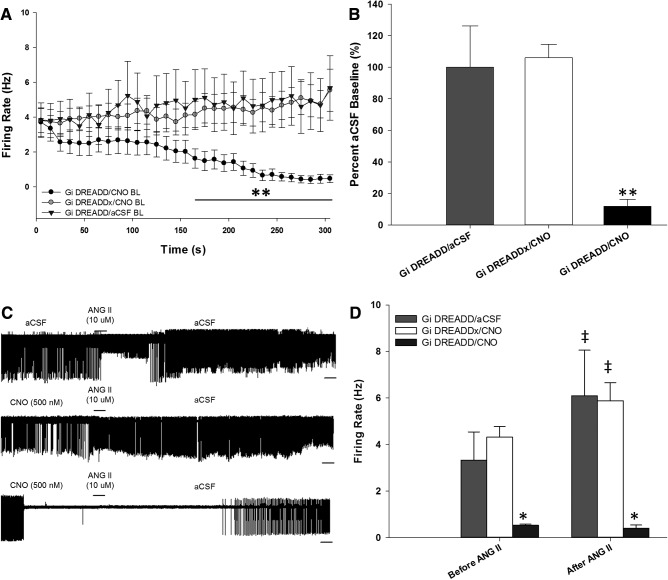
CNO-mediated inhibition blocks ANG II-induced excitation. ***A***, CNO bath application significantly inhibits basal firing rate of G_i_ DREADD neurons (*n* = 15 neurons, six rats), but does not affect spontaneous activity in control (*n* = 7 neurons, three rats) or G_i_ DREADD-unlabeled (G_i_ DREADDx) neurons (*n* = 15 neurons, six rats); **p < 0.050 compared to each group. ***B***, G_i_ DREADD neurons during CNO bath application reduced firing rate to 20% of percentage aCSF baseline but did not affect baseline of G_i_ DREADDx neurons. ***C***, Representative raw trace recordings of a G_i_ DREADD neuron during aCSF (control conditions) exposure (top), G_i_ DREADDx neuron (middle), and G_i_ DREADD neuron (bottom) with the two latter exposed to CNO during baseline. Scale bar, 10 s. ***D***, Focal ANG II application significantly increased firing rate of G_i_ DREADDx neurons during CNO exposure and G_i_ DREADD neurons during aCSF exposure; ‡*p* < 0.001. CNO-mediated inhibition blocked ANG II excitation of G_i_ DREADD neurons and displayed significantly reduced firing rate compared to G_i_ DREADDx neurons; *p < 0.001. Data are presented as mean and SEM.

Next, the effects of CNO on ANG II evoked responses in G_i_ DREADD MnPO neurons were tested. In these experiments, focally applied ANG II (10 μM) was administered during bath applications of CNO (500 nM). Bath application of CNO containing aCSF significantly reduced the spontaneous activity of G_i_ DREADD neurons (*n* = 15, six rats, two slices per rat) as compared to aCSF alone (*n* = 7, three rats, two slices per rat). In contrast bath application of CNO did not affect the activity of G_i_ DREADDx neurons from the same slices (*n* = 15, six rats, two slices per rat). In the G_i_ DREADD-positive neurons, this inhibition of spontaneous activity by CNO bath application was significant at 170 s after the start of CNO exposure and was maintained throughout the duration of the protocol (*F*_(2,39)_ = 5.577, *p* = 0.007, two-way repeated measures ANOVA; Fig. 3*A*,*B*). When analyzed as a percentage change from aCSF baseline, the activity of G_i_ DREADD cells was significantly reduced to 20% of baseline during CNO bath application, whereas G_i_ DREADDx cells were not affected (*F*_(58,1131)_ = 4.221, *p* < 0.001, two-way repeated measures ANOVA; Fig. 3*C*,*D*).

During aCSF bath application, focally applied ANG II significantly increased the firing rate of seven out of seven G_i_ DREADD neurons (*n* = 7, three rats, two slices per rat). The activity of G_i_ DREADDx neurons was increased by focal ANG II during CNO bath application (*F*_(3,66)_ = 840.408, *p* < 0.001, one-way ANOVA). However, CNO bath application blocked the responses of 10 out of 15 G_i_ DREADD-labeled neurons to focally applied ANG II (*p* = 0.258, Tukey’s *post hoc* test; Fig. 3*D*). There was no significant difference in baseline firing rates between G_i_ DREADD neurons before CNO exposure or G_i_ DREADDx neurons during CNO or aCSF exposure (*F*_(3,51)_ = 2.253, *p* = 0.093, one-way ANOVA).

### CNO significantly attenuates excitation produced by hypertonic saline in G_i_ DREADD-expressing neurons

**Figure 4.**
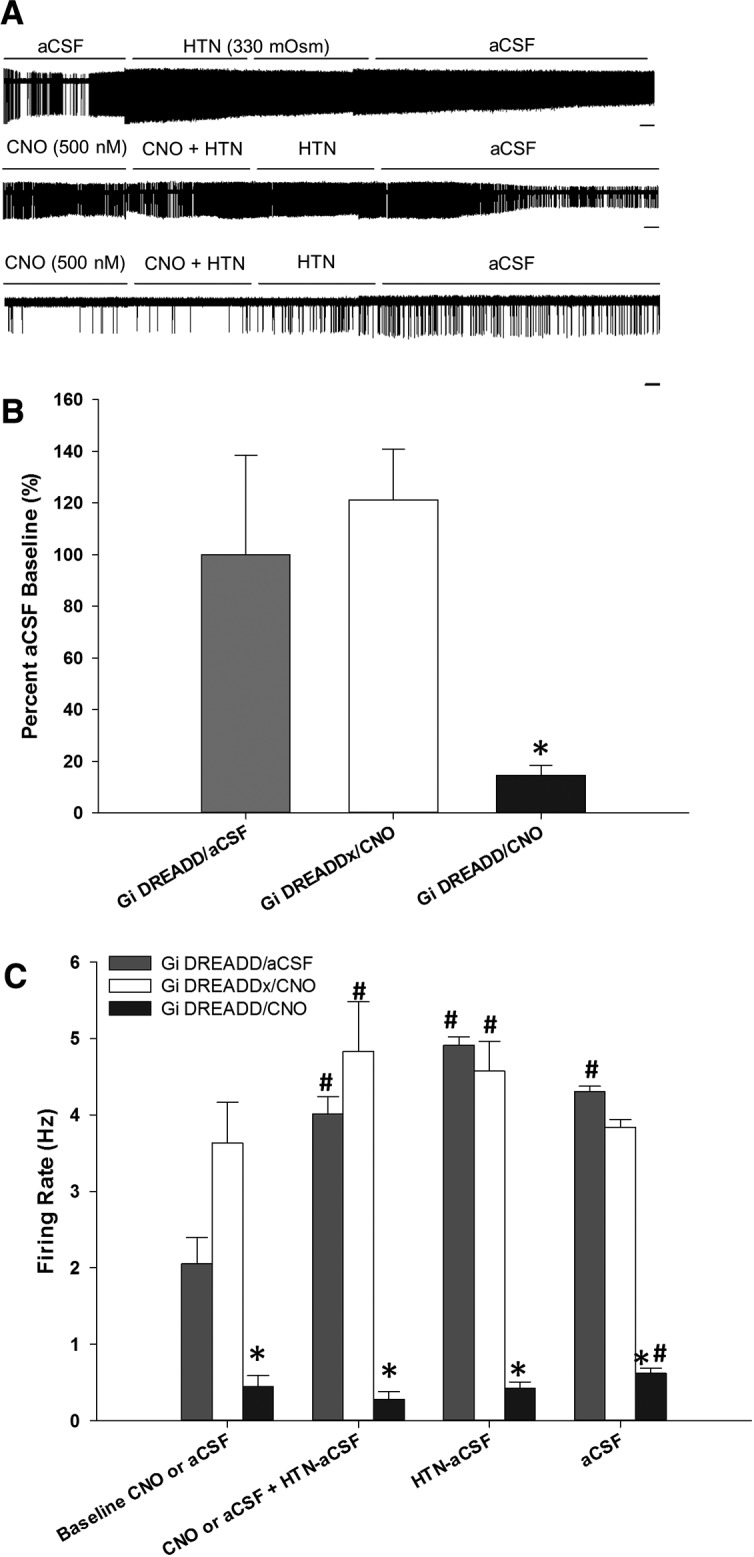
CNO-mediated inhibition attenuates firing rate during hyperosmotic challenges. ***A***, Representative raw trace recordings of a G_i_ DREADD neuron in the absence of CNO, using aCSF for control conditions (top), G_i_ DREADD-unlabeled (G_i_ DREADDx) neuron (middle), and G_i_ DREADD neuron (bottom), both latter neurons exposed to CNO. Scale bar, 10 s. Hypertonic aCSF, HTN-aCSF. ***B***, CNO significantly inhibits basal firing rate compared to percentage aCSF baseline of G_i_ DREADD neurons. ***C***, Hyperosmotic challenges significantly increased firing frequency of G_i_ DREADD neurons during aCSF exposure and G_i_ DREADDx neurons during CNO exposure (unaffected). CNO significantly attenuated firing rate of G_i_ DREADD neurons compared to G_i_ DREADDx neurons. There was an observed increase in firing rate in the absence of CNO during HTN-aCSF bath application, which became significant during aCSF bath application; *p < 0.001 compared to G_i_ DREADDx neurons exposed to CNO and G_i_ DREADD neurons during aCSF exposure; #*p* < 0.05 compared to time points within group. Data are presented as mean and SEM.

The effects of DREADD mediated inhibition on responses produced by bath application of HTN-aCSF (330 mOsm) was tested using MnPO neurons transfected with G_i_ DREADD or unlabeled cells in the same brain slices (G_i_ DREADDx). The activity of seven out of seven G_i_ DREADD neurons was significantly increased by HTN bath solution. As observed in the previous experiments, bath application of CNO decreased the basal activity of cells transfected with the G_i_ DREADD construct but did not affect the activity of G_i_ DREADDx cells (Fig. 4*A*,*B*).

Bath application of CNO did not influence the spontaneous activity or prevent increases in firing rate produced by HTN-aCSF in G_i_ DREADDx neurons compared to aCSF baseline (Fig. 4*A*). In contrast, CNO decreased spontaneous activity in G_i_ DREADD cells as compared to activity in normal aCSF (*F*_(2,41)_ = 112.004, *p* < 0.001, Holm–Sidak *post hoc* analysis; Fig. 4*A*). G_i_ DREADD neurons did not show a significant increase in firing rate associated with HTN-aCSF in the presence of CNO (Fig. 4*C*). Although the G_i_ DREADD cells appeared to be more active during bath application of CNO and HTN-aCSF, their average firing frequency was not different from CNO alone (Fig. 4*C*). The changes in firing rate of MnPO neurons was influenced by both the treatment and time as indicated by a statistically significant interaction (*F*_(89,2609)_ = 2.712, *p* < 0.001, two-way repeated measures ANOVA).

CNO bath application significantly attenuated changes in activity expressed as percentage of baseline activity in G_i_ DREADD neurons (*n* = 15, six rats, two slices per rat, *p* < 0.001, Tukey’s *post hoc* analysis). This was not the case for G_i_ DREADDx neurons (*n* = 14, six rats, two slices per rat) compared to aCSF bath applied G_i_ DREADD neurons (*n* = 7, two rats, two slices per rat; Fig. 4*A*,*B*). HTN-aCSF significantly increased the firing rate of unlabeled cells compared to baseline and aCSF recordings (*F*_(3,52)_ = 7.119, *p* < 0.001, one-way ANOVA; Fig. 4*C*). G_i_ DREADDx neurons had significantly increased firing rates when exposed to HTN-aCSF with or without CNO (*p* < 0.01, Tukey’s *post hoc* analysis; Fig. 4*C*). When the bath solution was changed to normal aCSF from HTN-aCSF, the activity of G_i_ DREADDx cells significantly decreased to a rate that was no different from CNO and normal aCSF (*p* = 0.301 baseline compared to aCSF; Fig. 4*C*). When G_i_ DREADD were exposed to HTN-aCSF without CNO, their firing rate significantly increased as compared to the aCSF baseline (*p* < 0.001 for all time points, Tukey’s *post hoc* analysis; Fig. 4*C*) and compared to CNO (*p* < 0.001 for all time points, Tukey’s *post hoc* analysis), but not when compared to G_i_ DREADDx neurons exposed to CNO.

In G_i_ DREADD neurons, there was a significant decrease in firing rate during CNO and HTN exposure compared to aCSF bath application (*F*_(3,56)_ = 30.084; *p* < 0.001, one-way ANOVA; *p* = 0.001, Tukey’s *post hoc* analysis). Although there was a trend for increased activity in G_i_ DREADD neurons exposed to HTN-aCSF after CNO was washed out (Fig. 4*C*), this change was not significantly different from CNO with HTN-aCSF (*p* = 0.917, Tukey’s *post hoc* analysis).

### Drinking responses

Based on the results of the electrophysiological studies, we tested the effects of intraperitoneal CNO (10 mg/kg) on drinking responses produced by ANG II (2 mg/kg, s.c.) in one group of rats and 3% HTN (1 ml/100-g bw, s.c.) in a separate group of rats. Control experiments using equal volumes of 0.9% saline (SAL) tests were also conducted for each group.

### Acute CNO-induced inhibition significantly attenuates ANG II-induced thirst responses

**Figure 5.**
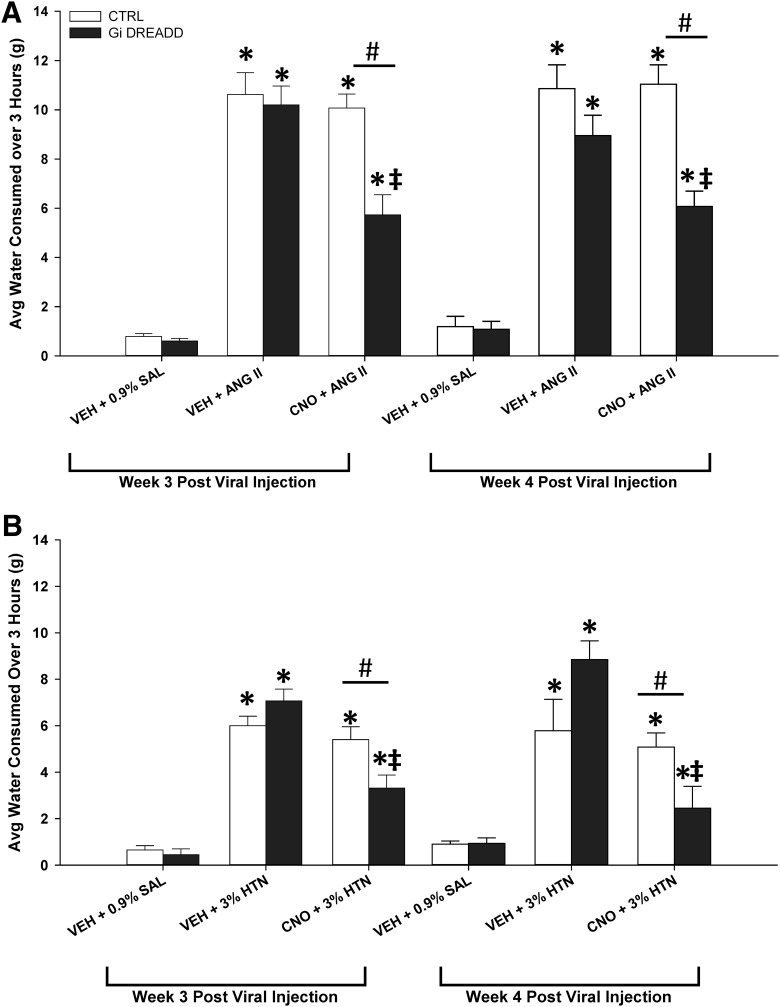
Acute MnPO inhibition attenuates evoked drinking responses. ***A***, ANG II significantly increased water consumption compared to volume control tests (VEH + 0.9% SAL) in both G_i_ DREADD and CTRL (VEH + ANG II) rats (*n* = 10 rats). CNO-mediated inhibition significantly attenuated this increase in G_i_ DREADD (CNO + ANG II) rats (*n* = 10 rats). ***B***, Hypertonic saline (3% HTN) significantly increased water consumption compared to VEH + 0.9% SAL tests in both G_i_ DREADD and CTRL (VEH + 3% HTN) rats (*n* = 6 rats) and CNO-mediated inhibition significantly attenuated this increase in G_i_ DREADD rats (*n* = 6 rats); *p < 0.005 compared to VEH + 0.9% SAL in respective group (***A*** or ***B***); ‡*p* = < 0.050 compared to VEH and ANG II (***A***) and 3% HTN (***B***) exposure in respective group; #*p* < 0.015 compared to CTRL + CNO + ANG II (***A***) and CTRL + CNO + 3% HTN (***B***). Data are presented as mean and SEM.

When rats were pretreated with CNO or VEH injections followed by ANG II treatment, there was an associated increase in water consumption by rats during the 3-h time period in both the G_i_ DREADD rats (*n* = 10) and CTRL rats (*n* = 10). Two-way repeated measures ANOVA analysis indicated a significant AAV variant dependent difference in water consumption (*F*_(1,18)_ = 18.925, *p* < 0.001, two-way repeated measures ANOVA; Fig. 5*A*) and treatment (VEH or CNO) received (*F*_(5,86)_ = 69.735, *p* < 0.001, two-way repeated measures ANOVA). There was no significant difference between groups during the 0.9% SAL tests [first treatment *p* = 0.989, last treatment *p* = 0.935, first treatment vs last treatment *p* = 0.999 (CTRL) and *p* = 0.998 (G_i_ DREADD)] or during VEH and ANG II test (*p* = 0.074) in the first treatment. During the CNO and ANG II test, however, the G_i_ DREADD group had approximately a 50% reduction in water consumed over the 3-h period compared to the CTRL group (*p* < 0.001, first treatment). This was also the case compared to the VEH and ANG II exposure (*p* = 0.005, first treatment). Independent of VEH or CNO treatment, ANG II still resulted in significantly elevated drinking response compared to physiologic saline volume control studies (all groups, *p* < 0.001, Tukey’s *post hoc* analysis).

One week later, ANG II significantly increased drinking behavior in the same manner compared to 0.9% SAL tests (all groups *p* < 0.001, Tukey’s *post hoc* analysis). There was no significant difference in thirst response between G_i_ DREADD or CTRL groups during VEH and ANG II exposure (*p* = 0.292, last treatment). During the CNO and ANG II treatment, however, the G_i_ DREADD group had approximately a 50% reduction in water consumed over the 3-h period compared to the CTRL group, similar to what was observed in the first treatment (*p* < 0.001, last treatment).

### Acute CNO-induced inhibition significantly attenuates hypertonic saline-induced thirst

A separate cohort of rats were injected in the MnPO with either G_i_ DREADD (*n* = 6 rats) or CTRL (*n* = 6 rats) virus and used to test whether or not CNO pretreatment would block the effects of 3% HTN injections on drinking behavior. As in the ANG II drinking studies, there was a significant interaction between AAV variant, pretreatment and 3% HTN exposure (*F*_(5,71)_ = 3.317, *p* = 0.015, two-way repeated measures ANOVA; Fig. 5*B*). When either of the groups were pretreated with VEH followed by 3% HTN, rats drank a significant amount of water over the 3-h time period compared to 0.9% SAL tests (Tukey’s *post hoc* analysis, 0.9% SAL vs CTRL + VEH + 3% HTN *p* < 0.001, 0.9% SAL vs G_i_ DREADD + VEH + 3% HTN *p* < 0.001, CTRL + VEH + 3% HTN vs G_i_ DREADD + VEH + 3% HTN group *p* = 0.794; Fig. 5*B*). However, when groups were pretreated with CNO before 3% HTN, the drinking response of the rats injected with G_i_ DREADD were significantly attenuated as compared to their responses to VEH and 3% HTN treatment (Tukey’s *post hoc* analysis; 0.9% SAL vs CTRL + CNO + 3% HTN *p* < 0.001, 0.9% SAL vs DREADD + CNO + 3% HTN *p* < 0.001, CTRL + CNO + 3% HTN vs DREADD + CNO + 3% HTN *p* = 0.011; Fig. 5*B*). Rats were injected with the same volume of 0.9% SAL and this treatment did not significantly affect water intake between groups (*p* = 0.799, Tukey’s *post hoc* analysis; Fig. 5*B*).

After one week, the same rats were retested. Injections of HTN significantly increased water intake compared to 0.9% SAL tests (all groups, *p* < 0.001, Tukey’s *post hoc* analysis). There was no significant difference in water intake between groups during VEH and 3% HTN exposure (*p* = 0.319, last treatment). During the CNO and 3% HTN treatment, the G_i_ DREADD group had approximately a 50% reduction in water consumed over the 3-h period compared to the CTRL group, similar to what was observed during the first treatment (*p* = 0.028, last treatment).

### Arginine vasopressin responses

Two days after completing the last drinking tests, rats were injected with VEH or CNO (10 mg/kg, i.p.) followed by either ANG II (2 mg/kg, s.c.), 3% HTN (1 ml/100-g bw, s.c.) or 0.9% SAL (volume control) 30 min later, as described in the drinking tests. Blood samples were taken and analyzed for plasma AVP concentrations.

### CNO-induced inhibition significantly attenuates ANG II-induced plasma vasopressin release

**Figure 6.**
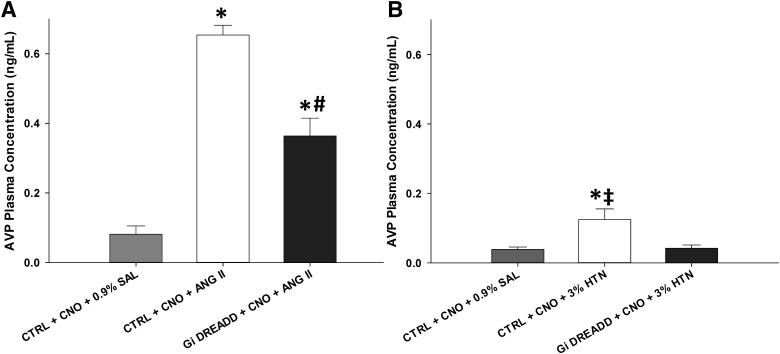
CNO-induced inhibition of CaMKIIa MnPO neurons significantly attenuates evoked increases of plasma AVP. ***A***, ANG II significantly increased plasma AVP concentration in CTRL rats (CTRL + CNO + ANG II), but this increase was significantly attenuated during CNO-mediated inhibition in G_i_ DREADD (G_i_ DREADD + CNO + ANG II) rats. ***B***, Hypertonic saline (3% HTN) significantly increased plasma AVP concentration, but this increase was blocked during CNO-mediated inhibition in G_i_ DREADD (G_i_ DREADD + CNO + 3%HTN) rats; *p < 0.02 compared to respective controls (CTRL + CNO + 0.9% SAL); ‡*p* < 0.050 compared to VEH and 3% HTN exposure; #*p* < 0.001 compared to CTRL + CNO + ANG II. Data are presented as mean and SEM.

The effects of DREADD inhibition in the MnPO on ANG II-induced AVP release was tested. Rats injected in the MnPO with the control vector (CTRL) that were pretreated with CNO and administered ANG II (CTRL + CNO + ANG II) had significantly increased plasma AVP (*F*_(2,23)_ = 44.963, *p* < 0.001, one-way ANOVA; Fig. 6*A*). This increase in plasma AVP associated with ANG II treatment was significantly decreased by CNO-induced inhibition in the G_i_ DREADD (G_i_ DREADD + CNO + ANG II) group (*p* < 0.001, Tukey’s *post hoc* analysis). However, the changes in plasma AVP observed in the G_i_ DREADD + CNO + ANG II were significantly increased compared to the vehicle control group (CTRL + CNO + 0.9% SAL) suggesting the ANG II-induced AVP release was significantly attenuated, but not reduced to control levels by MnPO inhibition (*p* < 0.001; Fig. 6*A*).

### CNO-induced inhibition blocks hypertonic saline-induced plasma vasopressin release

Plasma AVP concentration was significantly different between groups treated with 3% HTN compared to 0.9% SAL (*F*_(2,15)_ = 6.443, *p* = 0.010, one-way ANOVA; Fig. 6*B*), as well. Subcutaneous injection of 3% HTN significantly increased plasma AVP levels in the CTRL + CNO + 3% HTN from the vehicle controls (CTRL + CNO + 0.9% SAL) group (*p* = 0.016; Tukey’s *post hoc* analysis). Consistent with the ANG II plasma AVP results, CNO pretreatment significantly attenuated this increase in the G_i_ DREADD + CNO + 3% HTN group (*p* = 0.008, Tukey’s *post hoc* analysis). There was no significant difference in plasma AVP concentrations between the CTRL + CNO + 0.9% SAL and the G_i_ DREADD + CNO + 3% HTN group (*p* = 0.893, Tukey’s *post hoc* analysis).

### Functional neuroanatomy

**Figure 7.**
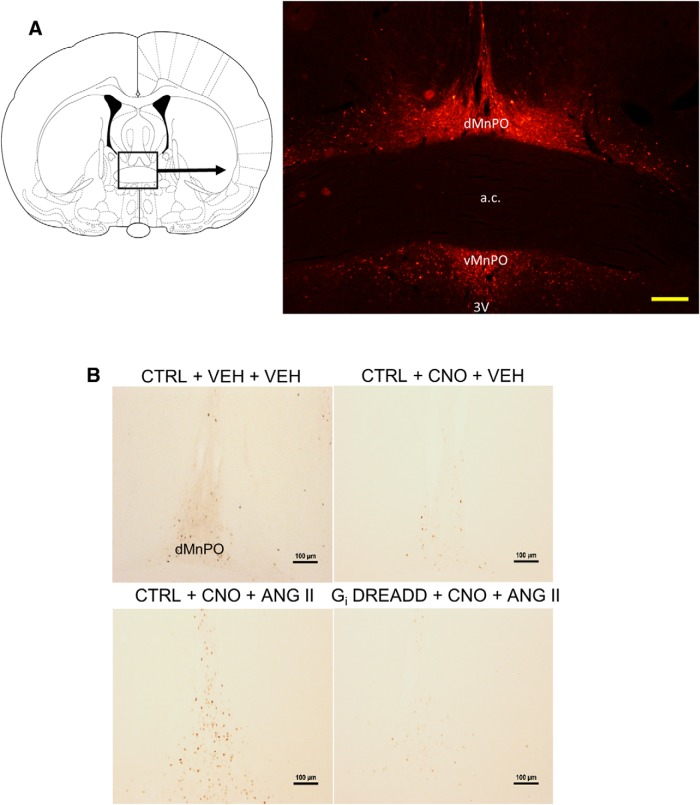
ANG II significantly increases Fos expression in the MnPO in control virus-injected rats, but is blocked during CNO-mediated inhibition in G_i_ DREADD-injected rats. ***A***, Diagram showing representative mCherry labeling from AAV transfection in the MnPO. Scale bar, 250 µm. Third ventricle, 3V; anterior commissure, a.c.; dorsal MnPO, dMnPO; ventral MnPO, vMnPO. ***B***, Representative Fos staining in the dMnPO of control virus-injected (CTRL) rats treated with CNO vehicle (VEH) and ANG II vehicle (0.9% SAL; *n* = 4, two to four sections per rat; upper left panel), CTRL rats treated with CNO and 0.9% SAL (*n* = 4, two to four sections per rat; upper right panel), CTRL rats treated with CNO and ANG II (*n* = 10, two to four sections per rat; lower left panel), and G_i_ DREADD-injected rats treated with CNO and ANG II (*n* = 10, two to four sections per rat; lower right panel). Scale bar, 100 µm.

Brains harvested from perfusions were analyzed for Fos and mCherry expression associated with the AAVs MnPO injections were verified by detecting the presence of mCherry immunofluorescence (Fig. 7*A*). Rats included in the study had mCherry expression isolated to the MnPO. Rats with injections or mCherry labeling outside of the MnPO were excluded from the study.

### CNO-induced inhibition during ANG II exposure significantly blocks CaMKIIa+ Fos expression in the MnPO

In rats treated with the control vector injections of CNO followed by subcutaneous injection of 0.9% saline (CTRL + CNO + 0.9% SAL) produced Fos staining in the MnPO that was not different from Fos staining associated with VEH injections paired with 0.9% saline subcutaneously (CTRL + VEH + 0.9% SAL; Fig. 7*B*; Table 1). Rats treated with the control vector and injected with CNO and ANG II (CTRL + CNO + ANG II) had a significant increase in Fos staining in the MnPO (*F*_(3,24)_ = 32.575, *p* < 0.001, one-way ANOVA; Fig. 7*B*; Table 1).

**Table 1.** Colocalization of ANG II-induced Fos expression with CaMKIIa neurons in the MnPO

Treatment	Total cFos+	Total CaMKIIa+	Total DL cells	% DL cFos+
Control CNO + 0.9% SAL	21.0 ± 10.0	182.8 ± 54.8	8.8 ± 1.25	50.2 ± 17.9%
Control VEH + 0.9% SAL	25.8 ± 7.8	181.5 ± 37.5	15.1 ± 3.4	60.0 ± 4.8%
Control CNO + ANG II	79.8 **±** 8.7**	195.0 ± 57.6	44.0 **±** 7.8**	54.4 ± 6.7%
G_i_ DREADD CNO + ANG II	41.9 ± 7.2	206.5 ± 41.0	2.9 ± 1.0	6.5 **±** 2.9%**

ANG II exposure significantly increases Fos expression in the MnPO, specifically in CaMKIIa-positive neurons. CNO-mediated inhibition significantly attenuated ANG II-induced Fos expression in the MnPO in G_i_ DREADD-injected rats, with inhibition of the CaMKIIa neuronal phenotype; Bold text and **p < 0.001 compared to all groups. Data are presented as mean ± SEM.

In the G_i_ DREADD + CNO + ANG II group (*n* = 10, two to four MnPO sections per rat), the increase in Fos staining in the MnPO was significantly decreased as compared to the CTRL + CNO + ANG II group (*n* = 10, two to four MnPO sections per rat; Tukey’s *post hoc* analysis, *p* < 0.001) but not different from the Fos staining observed in CTRL + VEH + 0.9% SAL (*p* = 0.170) or CTRL + CNO + 0.9% SAL(*p* = 0.531) rats. Fos staining in the MnPO was significantly increased in CTRL + CNO + ANG II rats compared to either the CTRL + VEH + 0.9% SAL (*p* < 0.001) or CTRL + CNO + VEH (*p* < 0.001) rats. Elevated MnPO Fos expression was blunted in G_i_ DREADD + CNO + ANG II rats (*p* < 0.001). There was no significant difference between CTRL groups treated with either VEH or CNO (*p* = 0.916) and 0.9% SAL.

### CaMKIIa neuron inhibition in the MnPO significantly decreases ANG II-evoked effects in downstream nuclei

As expected, ANG II significantly increased Fos staining in CaMKIIa^+^ neurons (*F*_(3,12)_ = 17.430, *p* < 0.001, one-way ANOVA). The numbers of Fos and CaMKIIa labeled in MnPO were significantly higher in the CTRL + CNO + ANG II group compared to the CTRL + VEH + 0.9% SAL (*n* = 4, two to four MnPO sections per rat; *p* < 0.001) and CTRL + CNO + 0.9% SAL (*n* = 4, two to four MnPO sections per rat; *p* = 0.003) groups. About half (54.4%) of the Fos-positive cells in the MnPO were also CaMKIIa-positive in the CTRL + CNO + ANG II rats. The percentages of Fos and CaMKIIa-positive cells were 50.2% and 60.0% for CTRL + CNO + 0.9% SAL and CTRL + VEH + 0.9% SAL rats, respectively. The increase in Fos and CaMKIIa staining cells associated with ANG II was significantly attenuated in the G_i_ DREADD+ CNO + ANG II rats (*n* = 4, two to four MnPO sections per rat; *p* < 0.001; Fig. 7*B*). The percentage of Fos-positive cells that were also CaMKIIa-positive was reduced to 6.5%. This result indicates that over 90% of the remaining Fos-positive cells in the MnPO of rats injected with G_i_ DREADD were not CaMKIIa-positive cells, which is significantly greater than the other three treatment groups (*F*_(3,12)_ = 6.010, *p* = 0.010, one-way ANOVA; Tukey’s *post hoc* analysis, all *p* < 0.001; Fig. 7*B*; Table 1). Since these cells do not appear to express CaMKIIa, they would not have been transfected with the AAV vector used in this study. There was no significant difference in CaMKIIa-positive neurons between any of the four groups (*F*_(3,12)_ = 0.0901, *p* = 0.964, one-way ANOVA).

**Figure 8.**
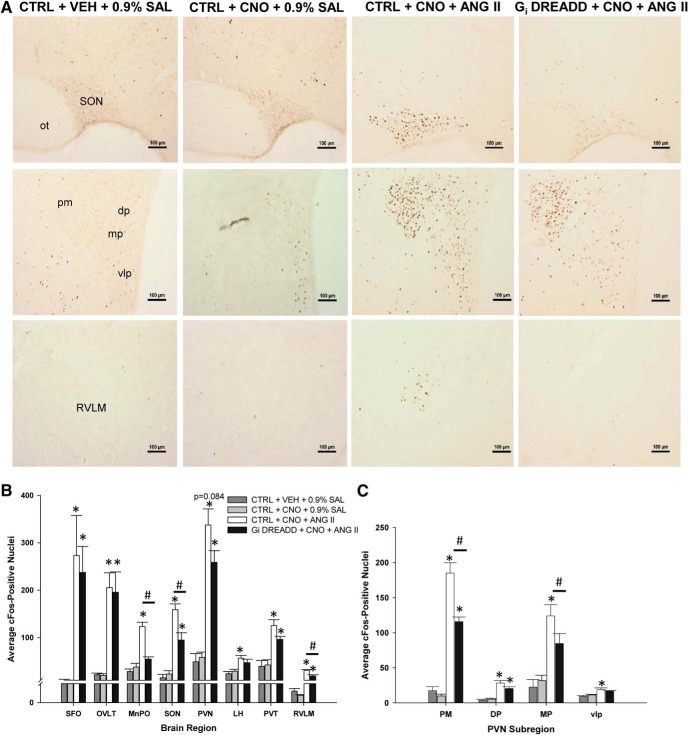
ANG II significantly increases Fos expression in regions downstream of the MnPO compared to controls and is blunted during CNO-induced inhibition in G_i_ DREADD-injected rats. ***A***, Representative Fos staining in the SON (top row), PVN (middle row), and RVLM (bottom row) for each group tested. Scale bar, 100 µm. Supraoptic nucleus, SON; optic tract, ot: paraventricular nucleus, PVN; posterior magnocellular, PM; dorsal parvocellular, DP; medial parvocellular, MP; ventrolateral parvocellular, vlp; rostral ventral lateral medulla, RVLM. ***B***, ANG II significantly increases Fos expression in the SFO, OVLT, PVN, and PVT but this increase was significantly attenuated during CNO-mediated inhibition in the MnPO, SON, LH, and RVLM. Subfornical organ, SFO; organum vasculosum of the lamina terminalis, OVLT; paraventricular thalamus, PVT; lateral hypothalamus, LH.***C***, ANG II significantly increases Fos staining in cardiovascular and neuroendocrine-regulating regions of the PVN, but is significantly attenuated by CNO-mediated inhibition in the PM and MP subregions; *p < 0.005 compared to VEH (CTRL + VEH + 0.9% SAL) and CNO (CTRL + CNO + 0.9% SAL) controls; #*p* < 0.001 G_i_ DREADD-injected rats compared to control virus-injected rats treated with CNO and ANG II (G_i_ DREADD + CNO + ANG II and CTRL + CNO + ANG II, respectively). Data are presented as mean and SEM.

G_i_ DREADD-mediated inhibition of the MnPO also influenced Fos staining associated with ANG II in regions connected to the MnPO (G_i_ DREADD + CNO + ANG II group: *n* = 6–10, two to four sections per nucleus per rat; CTRL + CNO + ANG II group: *n* = 4–10, two to four sections per nucleus per rat). In rats injected with the CTRL AAV vector and pretreated with CNO, ANG II significantly increased Fos staining in each region that we examined (Fig. 8). However, the effects of G_i_ DREADD MnPO inhibition on ANG II induced Fos staining varied as a function of region. In the rats injected with G_i_ DREADD, CNO pretreatment did not significantly affect ANG II induced Fos staining in SFO, OVLT, LH, or PVT (Fig. 8*B*).

In other regions, G_i_ DREADD-mediated inhibition of the MnPO did influence Fos staining. In the SON, CNO pretreatment in rats injected in the MnPO with G_i_ DREADD was associated with a significant decrease in ANG II-induced Fos (*F*_(3,24)_ = 19.328, *p* < 0.001, one-way ANOVA; Fig. 8*A*,*B*). Fos staining in the SON of CTRL + CNO + ANG II rats was significantly higher as compared to all of the other groups (Tukey’s *post hoc* analysis, all *p* < 0.001; Fig. 8*A*,*B*). Similar results were seen in the rostral ventrolateral medulla (RVLM) (Fig. 8*A*,*B*). ANG II injections significantly increased Fos staining in the CTRL + CNO + ANG II rats but not in the G_i_ DREADD + CNO + ANG II rats (*F*_(3,16)_ = 29.480, *p* < 0.001, one-way ANOVA).

In the PVN, CNO and ANG II significantly increased Fos staining in CTRL rats and there was a statistical trend for decreased Fos staining in the G_i_ DREADD + CNO + ANG II as compared to CTRL + CNO + ANG II (*F*_(3,24)_ = 18.302, *p* < 0.001, one-way ANOVA; Tukey’s *post hoc* analysis, *p* = 0.084). However, significant effects of G_i_ DREADD-mediated inhibition on Fos staining were observed in specific subregions of the PVN (Fig. 8*C*). For example, the posterior magnocellular (PM) part of PVN Fos staining was significantly increased by ANG II in CTRL rats while CNO significantly decreased Fos staining associated with ANG II in G_i_ DREADD rats (*F*_(3,22)_ = 49.423, *p* < 0.001, one-way ANOVA; Tukey’s *post hoc* analysis, CTRL + CNO + ANG II; *p* < 0.001 from all other groups). Fos staining in the PM of G_i_ DREADD + CNO + ANG II rats was still significantly higher when compared to Fos staining in rats pretreated with either CNO or VEH followed by 0.9% SAL (Tukey’s *post hoc* analysis; *p* = 0.05 and *p* = 0.097, respectively). DREADD-mediated inhibition of the MnPO influenced Fos staining in the medial parvocellular region (MP) of the PVN as well. In the MP part of the PVN, Fos staining associated with CNO and ANG II injections in CTRL rats was significantly greater than Fos staining observed in any of the other groups (*F*_(3,22)_ = 13.789, *p* < 0.001, one-way ANOVA; Tukey’s *post hoc* analysis, all *p* < 0.001). The Fos staining in the MP of G_i_ DREADD + CNO + ANG II rats was not significantly different from the CTRL rats pretreated with either CNO or VEH followed by 0.9% SAL (Tukey’s *post hoc* analysis; *p* = 0.097 and *p* = 0.050, respectively). This suggested that in the MP region of the PVN, inhibition of the MnPO reduced ANG II-induced Fos staining comparably to control levels. Similar results were observed in the ventrolateral parvocellular region (vlp). Fos staining was increased in CTRL + CNO + ANG II rats and this increase was significantly different from all of the other groups (*F*_(3,22)_ = 3.734, *p* = 0.026, one-way ANOVA). Fos staining in the dorsal parvocellular part of the PVN was increased by ANG II (*F*_(3,22)_ = 11.903, *p* < 0.001, one-way ANOVA) but CNO-induced inhibition did not significantly decrease it (CTRL + CNO + ANG II vs G_i_ DREADD + CNO + ANG II, *p* = 0.258, Tukey’s *post hoc* analysis).

### CNO-induced inhibition in the MnPO and Fos staining associated with 3% HTN

**Figure 9.**
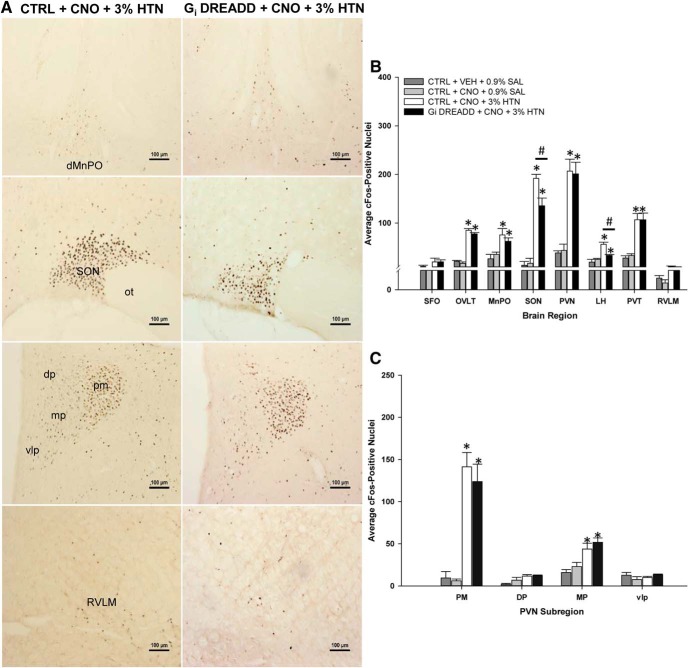
Hypertonic saline significantly increases Fos expression in the MnPO and in regions downstream, but is attenuated during CNO-induced inhibition in G_i_ DREADD-injected rats. ***A***, Representative Fos staining in control virus-injected (CTRL) rats (*n* = 6, two to four sections per rat; left) and G_i_ DREADD rats (*n* = 6, two to four sections per rat; right), treated with CNO and 3% HTN; dMnPO (top row), SON (second row), PVN (third row), RVLM (bottom row). ***B***, 3% HTN significantly increases Fos expression in the OVLT, MnPO, PVN, and PVT, but this increase was significantly attenuated during CNO-mediated inhibition in the SON and LH. Scale bar, 100 µm. Subfornical organ, SFO; organum vasculosum of the lamina terminalis, OVLT; paraventricular thalamus, PVT; lateral hypothalamus, LH. ***C***, 3% HTN significantly increases Fos staining in cardiovascular and neuroendocrine-regulating regions of the PVN, the PM and MP subregions; *p < 0.005 compared to VEH (CTRL + VEH + 0.9% SAL) and CNO (CTRL + CNO + 0.9% SAL) controls; #*p* < 0.001 G_i_ DREADD rats compared to CTRL rats treated with CNO and 3% HTN (G_i_ DREADD + CNO + 3% HTN and CTRL + CNO + 3% HTN, respectively). Data are presented as mean and SEM.

In rats treated with the control vector, Fos staining in the MnPO was significantly increased by 3% HTN (CTRL + VEH + HTN and CTRL + CNO + HTN; *F*_(3,16)_ = 4.982, *p* = 0.002, one-way ANOVA; Fig. 9*A*,*B*). Half of the cells that were Fos-positive were also positive for CaMKIIa (Table 2), suggesting that HTN affected cells that were not transfected with the vector.

**Table 2.** Colocalization of hypertonic saline-induced Fos expression with CaMKIIa neurons in the MnPO

Treatment	Total cFos+	Total CaMKIIa+	Total DL cells	% DL cFos+
Control CNO + 0.9% SAL	16.1 ± 3.7	226.4 ± 50.9	8.6 ± 2.2	63.3 ± 16.9%
Control VEH + 0.9% SAL	22.1 ± 4.0	202.9 ± 47.3	11.3 ± 2.1	74.6 ± 1.3%
Control CNO + 3% HTN	74.4 ± 17.6*	194.3 ± 59.4	30.0 ± 8.3**	49.5 ± 16.7%
G_i_ DREADD CNO + 3% HTN	**64.6** ± 6.5*	165.4 ± 22.1	3.4 ± 1.0	5.4 ± 1.9%**

Hypertonic saline exposure significantly increases Fos expression in the MnPO, not only in CaMKIIa-positive neurons, but also other neuronal phenotypes. CNO-mediated inhibition did not significantly attenuate total hypertonic saline-induced Fos expression in the MnPO in G_i_ DREADD-injected rats; Bold text and *p < 0.050 compared to vehicle controls; Bold text and **p < 0.001 compared to all groups. Data are presented as mean ± SEM.

Unlike our results with ANG II, Fos staining associated with 3% HTN in the MnPO was not significantly decreased by CNO pretreatment in G_i_ DREADD rats (Gi DREADD + CNO + HTN). Fos staining in the MnPO of the G_i_ DREADD + CNO + HTN group (*n* = 6 rats, two to four MnPO sections per rat) was significantly increased as compared to either of the CTRL groups injected with 0.9% SAL (CTRL + CNO + 0.9% SAL, *n* = 6 rats, two to four MnPO sections per rat; CTRL + VEH + 0.9% SAL, *n* = 6 rats, two to four MnPO sections per rat; *p* = 0.019 and *p* = 0.006, respectively). Fos staining in the G_i_ DREADD + CNO + HTN was not different from CTRL + CNO + 3% HTN rats (*p* = 0.893, Tukey’s *post hoc* analysis).

In contrast, the number of Fos-positive cells that were also labeled for CaMKIIa was significantly decreased (Table 2). As indicated above, half of the Fos-positive cells were CaMKIIa-positive in the CTRL + CNO + 3% HTN rats. In the G_i_ DREADD + CNO + 3% HTN rats, only 5% of the Fos-positive cells were also positive for CaMKIIa meaning 95% of the cells had a different phenotype and may have intrinsic osmotic sensitivity. There were no significant differences in the numbers CaMKIIa-positive neurons between any of the four groups (*F*_(3,12)_ = 0.287, *p* = 0.834, one-way ANOVA; Table 2). This suggests that the apparent lack of CNO inhibition on HTN-mediated Fos staining in the MnPO was due to activation of non-CaMKIIa-expressing cells.

### CaMKIIa neuron inhibition in the MnPO significantly decreases 3% HTN-evoked effects in select downstream nuclei

Analysis of Fos staining was performed in the same downstream nuclei as in the ANG II experiments above. While all of the regions except the SFO showed significant increases in Fos staining associated with the 3% HTN injections, only the SON and LH were affected by G_i_ DREADD-mediated inhibition of the MnPO (Fig. 9). In the SONs of CTRL + CNO + 3% HTN rats, Fos staining was significantly increased compared to all of the other groups (*F*_(3,16)_ = 52.418, *p* < 0.001, one-way ANOVA; all *p* < 0.001, Tukey’s *post hoc* analysis; Fig. 9*A*,*B*). In the SONs of G_i_ DREADD rats, CNO pretreatment significantly reduced Fos staining associated with 3% HTN as compared to CTRL + CNO + 3% HTN rats (*p* = 0.012, Tukey’s *post hoc* analysis) but did not reduce the staining to control levels (vs CTRL + VEH + 0.9% SAL, *p* < 0.001; vs CTRL + CNO + 0.9% SAL, *p* < 0.001).

Similar results were observed in the LH. Rats in the CTRL + CNO + 3% HTN group had significantly more Fos staining in the LH compared to the other three groups (*F*_(3,16)_ = 18.937, *p* < 0.001, one-way ANOVA; all *p* < 0.001, Tukey’s *post hoc* analysis; Fig. 9*B*). Fos staining in the LH of G_i_ DREADD + CNO + 3% HTN rats had significantly less Fos expression than the CTRL + CNO + 3% HTN-treated rats (*p* = 0.001, Tukey’s *post hoc* analysis; Fig. 9*B*) and significantly higher than both the 0.9% SAL-treated groups (both *p* < 0.001, Tukey’s *post hoc* analysis; Fig. 9*B*). For the remaining regions (OVLT, PVN, PVT, and RVLM) Fos staining associated with CNO and 3% HTN was not different between the CTRL virus and G_i_ DREADD-injected rats and both of these groups were significantly increased as compared to the two CTRL groups that received the 0.9% SAL injection (Fig. 9*A*,*B*). This result was also observed in the PM and MP regions of the PVN (Fig. 9*C*), while 3% HTN did not influence Fos staining in either the dorsal or vlp regions.

## Discussion

These studies tested the role of putative excitatory MnPO neurons in behavioral and physiologic responses produced by peripheral administration of ANG II or 3% HTN. The virally-mediated chemogenetic inhibition was employed in these studies to remotely and selectively reduce the activity of CaMKIIa-expressing MnPO neuronal population in the least invasive manner. These experiments showed that acutely inhibiting the CaMKIIa-expressing neurons in the MnPO significantly attenuated water intake and vasopressin release produced by either ANG II or 3% HTN. The results, however, suggest several differences in how the MnPO contributes to these responses.

In previous studies of the lamina terminalis and its role in water consumption, the SFO and OVLT have been linked to body fluid homeostasis. Studies performed in sheep using electrolytic lesions placed along the ventral lamina terminalis, including the OVLT, decreased water intake stimulated by cellular dehydration (McKinley et al., 1999). Chemogenetic activation of the CaMKIIa neuronal phenotype in the SFO has also been shown to influence drinking behavior (Nation et al., 2016). Studies performed by Oka et al. (2015) showed that 48-h water deprivation induced Fos immunoreactivity and were able to identify that in the SFO all of the neurons expressing Fos co-localized with CaMKIIa and nNOS. The SFO and the OVLT both project to the MnPO as well as other regions involved in body fluid homeostasis (McKinley et al., 2004). Recent studies conducted by Augustine et al. (2018) implicated the MnPO^nNOS^ neuronal phenotype in regulating drinking behavior stimulated by water deprivation and salt loading. The current study shows that CNO-induced inhibition of DREADD-transfected CaMKIIa MnPO neurons significantly attenuates extracellular and cellular thirst. The result of the Fos studies indicate, however, that the homeostatic responses could involve different pathways from the MnPO.

These studies specifically targeted CaMKIIa neurons in the MnPO, which as shown in the *in situ* hybridization studies, is highly co-localized with vGLUT2. This indicates that CaMKIIa neurons in the MnPO are primarily glutamatergic. This is an important consideration since the MnPO has been shown to contain cells that can stimulate or inhibit water intake (Oka et al., 2015). The results of the current study extend the observations of Oka et al. (2015) by demonstrating that water intake related to extracellular and cellular dehydration is mediated by putative excitatory neurons in MnPO.

The CaMKIIa MnPO neurons transfected by the virus were sensitive to both ANG II and HTN-aCSF. The design of the experiments prevented us from being able to test the same cells with both ANG II and HTN-aCSF. Previous studies have defined a set of sodium sensitive MnPO neurons that likely contribute to body fluid balance (Grob et al., 2004) and additionally characterized ANG II-sensitive MnPO neurons in earlier experiments, consistent with our findings (Bai and Renaud, 1998). The relationship between ANG II and HTN-aCSF in the regulation of the MnPO neurons will be the focus of future investigations.

The CNO-mediated inhibition of CaMKIIa-positive neurons in the MnPO completely blocked ANG II-induced excitatory responses. However, during osmotic challenges, CNO-mediated inhibition was only able to significantly attenuate osmotic-induced excitatory response, in contrast to the complete inhibition of ANG II-induced excitation. This is consistent with studies conducted by Grob et al. (2004) describing a specific MnPO neuronal phenotype with intrinsic sodium sensitivity. Recordings performed in the current experiments also verify that G_i_ DREADDx neurons, or MnPO neurons that do not express CaMKIIa, are also sensitive to ANG II and osmotic challenges (Grob et al., 2004).

Results from these Fos studies further support the electrophysiology findings and suggest that ANG II and 3% HTN mediated responses could involve different populations of CaMKIIa-expressing MnPO neurons. Fos immunohistochemistry and its co-localization with CaMKIIa in the MnPO was analyzed. Neurons in the MnPO both expressing CaMKIIa and Fos were significantly elevated in groups injected with the control virus and administered ANG II or 3% HTN. During DREADD-mediated inhibition using CNO, however, there were significantly reduced numbers of CaMKIIa-positive cells that were also positive for Fos in the MnPO. While this led to a significant decrease in the total number of Fos-positive cells associated with ANG II, the overall decrease in the MnPO was not significant after hypertonic saline. This could be related to the electrophysiology data showing that CNO did not completely block the hyperosmotically-stimulated excitation as strongly as the ANG II responses in the G_i_ DREADD-transfected neurons.

The differences observed for Fos staining in the MnPO could be related to the nonspecific effects of the hypertonic saline injections. While hypertonic saline is widely used as an osmotic stimulus, Fos staining associated with its administration may be a result of visceral pain related to the route of injection, and therefore, not specific. However, a study conducted by Xu et al. (2003) compared Fos staining produced by intraperitoneal versus intravenous administration of 2.0 M NaCl in several of the regions included in the current study (MnPO, OVLT, PVN, and SON). They concluded that the increases in Fos staining observed in areas such as the MnPO were related osmotic stimulation and fluid balance and not nociception. Similar conclusions were made about Fos staining in the LH produced intraperitoneal administration of 1.5 M NaCl (Pirnik et al., 2004). It should be noted that the concentration of NaCl used in the current study are approximately four times lower than the concentration used by Xu et al. (2003) and that this study used subcutaneous injections to avoid producing visceral pain associated with intraperitoneal injections. Therefore, the Fos staining produced by hypertonic saline is more likely related to osmotic stimulation but the contribution of nociceptive stimulation at the injection site cannot be ruled out.

There were also observed differences in Fos staining in several other regions that receive projections from the MnPO that were stimulus dependent. In rats administered ANG II, CNO-induced inhibition of G_i_ DREADD was associated with significant decreases in Fos staining in the SON, PVN, and RVLM. When rats were injected with hypertonic saline, G_i_ DREADD mediated inhibition of the MnPO significantly attenuated Fos staining in the SON and LH but not the PVN or RVLM. The central effects of ANG II and hypertonic saline are mediated by CVOs (Ferguson, 2014). These regions project not only to the MnPO but also to the SON, PVN, and LH (McKinley et al., 2004, 2015; Ferguson, 2014). This creates a series of redundant pathways from the lamina terminalis to regions contributing to hormone release and water intake. Our results suggest that, despite these possible redundancies, the MnPO significantly contributes to the activation of SON, PVN, and LH in a stimulus-dependent manner.

As shown in these studies, G_i_ DREADD inhibition of the MnPO was associated with significant decreases in Fos staining in the SON after either ANG II or 3% HTN. These results are consistent with observed reductions in AVP release associated with G_i_ DREADD inhibition of the MnPO during ANG II or 3% HTN exposure, with ANG II inducing a greater release of AVP than 3% HTN between groups. This may be due to how each stimulus induces its effects in the local circuitry. In ANG II-treated rats, G_i_ DREADD inhibition of the MnPO reduced Fos staining in the PVN and RVLM, while these regions were not influenced by MnPO inhibition in hypertonic saline-treated rats. Fos staining in the LH that was associated with hypertonic saline was significantly decreased by G_i_ DREADD inhibition. Recent studies suggest that the PVN, PVT, and LH receive projections from the MnPO that are related to water intake (Leib et al., 2017). Results from the current study are generally consistent with these findings and suggest that these pathways might be differentially regulated by extracellular dehydration versus cellular dehydration (Fitzsimons, 1972). Differences in MnPO neurons that contribute to extracellular versus cellular was first proposed based on the results of studies using excitotoxins (Cunningham et al., 1991). The current results provide evidence that MnPO neurons mediating water intake associated with extracellular dehydration project to the PVN while MnPO neurons participating in cellular thirst project to the LH. These data also suggest that there are MnPO neurons participating in both extracellular and cellular dehydration that project to the SON.

Findings from the current study also demonstrate that Fos expression was significantly decreased in the RVLM during ANG II but not hypertonic saline exposure during G_i_ DREADD-mediated inhibition. The RVLM contains sympathetic motor neurons that contribute to blood pressure regulation. These results suggest that the MnPO may contribute to increases in sympathetic tone related to activation of the renin-angiotensin system but not high salt. This is consistent with previous studies on the role of the lamina terminalis in ANG II-dependent models of hypertension (Ployngam and Collister, 2007; Collister et al., 2014; Shell et al., 2016). The decreased Fos staining in the RVLM could be related to the decreased Fos expression observed in the PVN, which has also been linked to hypertension (Llewellyn et al., 2012; Basting et al., 2018). Lesions of the AV3V have been shown prevent or reverse several ANG II dependent models of hypertension (Brody et al., 1978). The results of the current study suggest that CaMKIIa neurons in the MnPO that project to the PVN could contribute to this aspect of AV3V function.

Interestingly, ANG II was much more effective at inducing differential Fos expression, water intake, and AVP release compared to 3% HTN. Studies by Fitzsimons showed that water consumption associated with hypovolemic dehydration can result in a greater volume of water intake as compared to cellular dehydration of similar magnitude (Fitzsimons, 1998). While it is difficult to determine the equivalence of extracellular versus cellular dehydration, the differences in the magnitude of the response could be related to the position of the doses used in their respective dose-response curves.

It is important to note how increases in arterial pressure associated with ANG II may alter its dipsogenic effects. Early studies addressing ANG II in relation to arterial pressure and thirst have been conducted. Rettig and Johnson (1986) studied the role of ANG II, among other stimuli, on drinking in sinaortic denervated rat models to hypovolemia, and found no effect on water intake. In other studies conducted by Evered et al. (1988), the interaction between the dipsogenic and pressor effects of ANG II at various concentrations were tested using diazoxide, a vasodilator, to counteract the increases in blood pressure (Evered et al., 1988). It was concluded from these studies that intravenous infusions of ANG II in water-replete rats stimulate thirst in a dose-dependent manner, but water intake may be attenuated due to the concurrent increase in blood pressure. The rise in arterial pressure may also produce increases in urinary water and solute excretion, resulting in dehydration that could eventually contribute to thirst as well (Evered et al., 1988). Most physiologic situations characterized by activation of the renin-angiotensin system include hypovolemia and hypotension. In this context, the increase in blood pressure produced by ANG II may allow the rats to be behaviorally competent enough to ingest water (Evered et al., 1988).

In summary, these studies used ANG II and hypertonic saline injections to simulate aspects of extracellular and cellular dehydration to better understand the contribution of the MnPO to the integrative physiology of body fluid homeostasis. Both of these stimuli act through CVOs to affect the MnPO, Additionally, hypertonic saline can directly influence the activity of sodium sensitive MnPO neurons. The chemogenetic inhibition of putative excitatory MnPO neurons inhibited vasopressin release and drinking behavior produced by either ANG II or hypertonic saline. In contrast, the effects of inhibiting CaMKIIa MnPO neurons was associated with different patterns of Fos staining. Fos staining in the SON, PVN, and RVLM associated with ANG II injections was significantly decreased by chemogenetic inhibition of the MnPO. After injections of hypertonic saline, MnPO inhibition affected the LH and SON. Thus, extracellular and cellular dehydration appear to influence different populations of CaMKIIa-expressing MnPO neurons based on their afferent projections but can result in converging behavioral outcomes.

## Synthesis

Reviewing Editor: Christian Lüscher, University of Geneva Decisions are customarily a result of the Reviewing Editor and the peer reviewers coming together and discussing their recommendations until a consensus is reached. When revisions are invited, a fact-based synthesis statement explaining their decision and outlining what is needed to prepare a revision will be listed below. The following reviewer(s) agreed to reveal their identity: Andrew Allen.

In general, while potentially of interest, both referees agreed that the key concern relates mostly to the description of the results. This is poorly organized with a lack of logic to the order of the presentation. It is particularly evident in the section on in vivo responses to Ang II, but all sections could receive attention. As far as I can tell the data are all presented, but the lack of clarity in presentation makes comparison between groups difficult.

Major:

1.) It is reasonable to use systemic administration of ANGII as a stimulus to drive volemic thirst; however, doing so has the disadvantage of increasing blood pressure in addition to eliciting thirst. Under most circumstances, this would not occur as the elevated ANGII that accompanies volume depletion would maintain blood pressure. Some discussion of how thirst driven by systemic administration of exogenous ANGII differs from thirst driven by actually volume depletion seems warranted.

2.) Please provide the volume of blood that was collected via cardiac puncture as well as the volume of plasma that was used for the AVP RIA.

3.) Much on the manuscript centers on how chemogenetic inhibition alters neuronal activation of virally transformed neurons in the MnPO and its target effector nuclei; however, the methods for image capture and analysis are not provided. This description is important for evaluating the validity of the results from the functional neuroanatomical experiments. Along these lines, if I am understanding the methods correctly, Fos labeling used DAB immunohistochemistry but virally transfected neurons were identified via anti-mCherry IHC (i.e. amplified via Cy3 secondary). If this is correct, then co-localization counts were done using a combination of light and fluorescent microscopy. This is unorthodox as co-localization is usually performed on optical sections through the z plane obtain from a confocal fluorescence microscope. Please provide an explanation of how this was accomplished.

4.) Evaluating the number of CAMKIIa neurons in the MnPO that express VGLUT2 mRNA is not ideal for determining how many virally transformed neurons in the MnPO are glutamatergic. Why wasn't this analysis conducted on rats delivered the virus? Along these lines, Figure 1 on does show that in the MnPO that many CAMKIIa neurons are glutamatergic; however, it also suggests that some are not. Given that the dogma is that CAMKIIa is a marker for forebrain glutamatergic neurons, how do the authors interpret these results?

5.) On page 15, “Focal CNO application did not significantly influence the spontaneous activity of neighboring, unlabeled neurons...” This is an important statement as it would imply that CAMKIIa neurons in the MnPO do not make local connections. How was this determined? Did the authors examine spontaneous firing rate, membrane potential or the frequency/magnitude of EPSCs?

6.) More details are need for the experiments evaluating the effect of ANGII or hypertonic saline on the firing rates of virally transformed MnPO neurons. Of the neurons samples in each conditions, how many responded to ANGII or hypertonic saline? In other words, what can be said about how many CAMKIIa neurons in the MnPO respond to ANGII or hypertonic saline?

This is poorly organized with a lack of logic to the order of the presentation. It is particularly evident in the section on in vivo responses to Ang II, but all sections could receive attention. As far as I can tell the data are all presented, but the lack of clarity in presentation makes comparison between groups difficult.

7.) The use of control to describe the non-DREADD expressing viral injected animals, or the vehicle-injected animals needs to be clearly thought through. It would be best to determine the term used for each, define it, and then use it consistently throughout. Similarly, with the chemogenetic receptor - please define and use either DREADD, or better, GiDREADD, at the first instance and use it consistently thereafter.

The DREADD approach promised the use of an inert ligand, CNO, to alter the activity of DREADD receptor expressing neurons. Several studies now show that is not the case and that CNO is likely having its central effects, after a peripheral injection, via conversion to clozapine.

Minor:

1.) Figure 7A. A large stitched confocal image of the entire coronal section would make a more compelling case for site specific injection into MnPO.

2.) The mention of the magnitude of each stimulus is warranted when discussing the differential Fos induction that occured following chemogenetic inhibition with ANGII or hypertonic saline administration. That is, ANGII was much better at driving thirst and AVP release than hypertonic saline and it is possible that this difference influenced Fos induction, etc.

3.) There are multiple errors in the manuscript that will likely be rectified as it is substantially revised as suggested above. These have not been identified in detail.

What is a loose-patch voltage clamp recording (page 9)?

The term “The parameter measured for action potential...” is used multiple times and could be consolidated into one use in the analysis section.

Please define the meaning of ‘peak firing rate’ - it is assumed this refers to the 10s bin with the lowest firing rate?

Please define the length of recording used to define the baseline.

How does the histogram in Fig 1D for baseline have error bars?

Please include in each figure legend that data are presented as mean +/- SEM.
